# P-1906. Early COVID-19 and Severity of Subsequent Omicron Infection in Ontario, Canada

**DOI:** 10.1093/ofid/ofae631.2067

**Published:** 2025-01-29

**Authors:** Caroline Kassee, Moe H Kyaw, Zoe Zhong, Altynay Shigayeva, Catherine Martin, Lubna Farooqi, Brenda Coleman, Wayne Gold, Christopher Kandel, Maria Major, Samira Mubareka, Srinivas Valluri, John M McLaughlin, Allison McGeer

**Affiliations:** Sinai Health System, Toronto, Ontario, Canada; Pfizer, New York, New York; Sinai Health System, University of Toronto, Toronto, Ontario, Canada; Sinai health, Toronto, Ontario, Canada; Pfizer Inc., Paris, Ile-de-France, France; Sinai Health System, University of Toronto, Toronto, Ontario, Canada; Sinai Health System, University of Toronto, Toronto, Ontario, Canada; University of Toronto, Toronto, ON, Canada; Toronto East General Hospital, Toronto, Ontario, Canada; Pfizer Canada, Kirkland, QC, Canada; Sunnybrook Health Sciences Centre, University of Toronto, Toronto, Ontario, Canada; Pfizer Inc, New York, New York; Pfizer, New York, New York; Mt. Sinai Hospital, Toronto, Ontario, Canada

## Abstract

**Background:**

As SARS-CoV-2 evolves, assessing changes in COVID-19 severity over time, and the impact of prior infection on repeat infection, is important. We determined whether developing COVID-19 early in the pandemic was associated with reduced severity of subsequent infection with Omicron sub-lineages.

**Figure:**
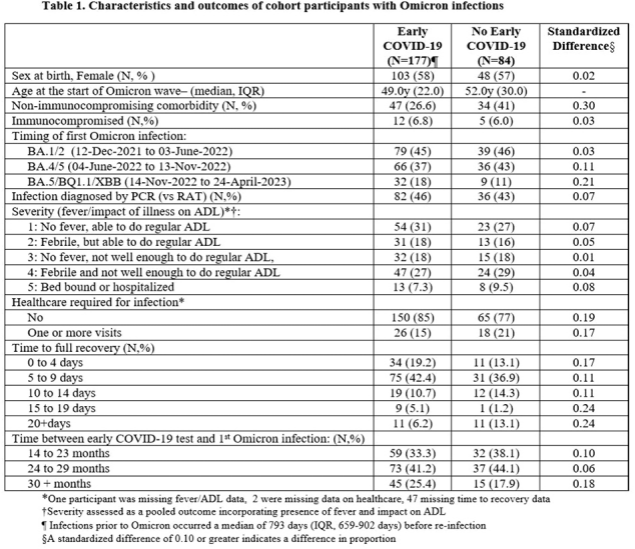

**Methods:**

We evaluated COVID-19 severity among patients infected during the Omicron wave. Severity was measured in 3 ways:(1) an ordinal measure combining activities of daily living (ADL) and presence of fever,(2) healthcare required (yes/no),(3) an ordinal measure of illness duration. We compared these outcomes in a study of age and time-period matched cohorts in Toronto, Canada: one with symptomatic COVID-19 between Mar 1 & Sep 30/2020 ('early COVID-19') and another who did not test positive for SARS-CoV-2 during the same period. Participants completed baseline, then biweekly surveys to identify SARS-CoV-2 infection episodes from Jan 2020 to Jan 2023. Multivariable binary and ordinal logistic regression models were used to construct ORs and 95% CI for impact of early COVID-19 on severity, adjusted for social/demographic characteristics, comorbidities, COVID-19 vaccination status, and time from early COVID-19 to first Omicron infection.

**Figure:**
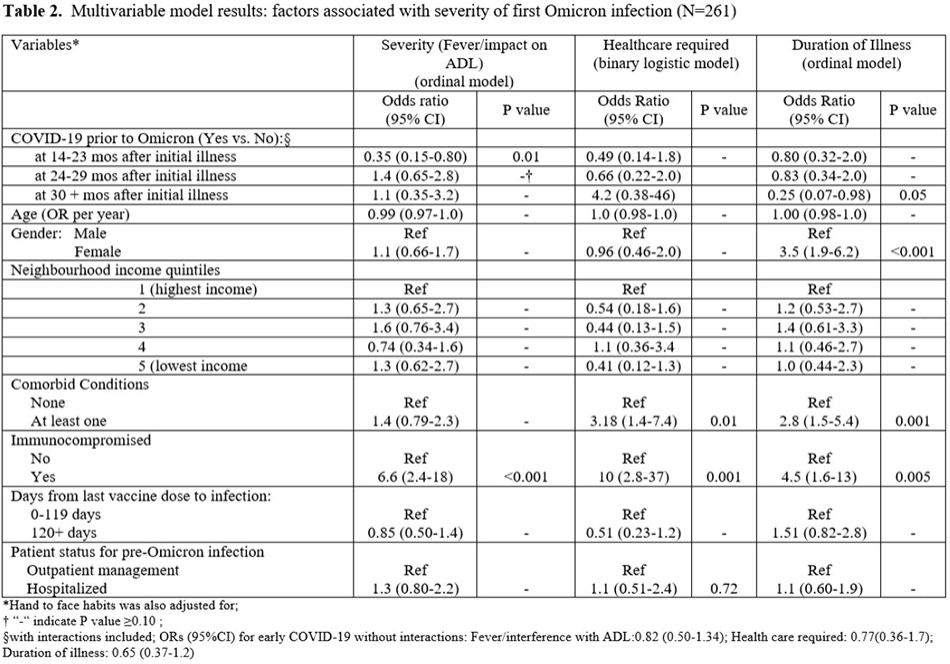

**Results:**

Of 261 participants with COVID-19 due to Omicron (Table 1), 177 had early COVID-19 at a median of 26 months prior. In adjusted analyses, those with early COVID-19 occurring < 24 months prior to their Omicron infection had lower odds of having severe Omicron-related illness; OR 0.35 (95%CI 0.15-0.80); with non-significant lower odds of requirement for healthcare (OR 0.49,95%CI 0.14-1.8) and illness duration (OR 0.80, 95%CI 0.32-2.0) (Table 2). Immunocompromise was associated with more severe illness based on all 3 outcomes; other non-immunocompromising comorbidities were associated with requiring healthcare and longer illness duration, and females reported longer illness duration (Table 2).

**Conclusion:**

Developing COVID-19 early in the pandemic was associated with reduced severity of first Omicron infection if it occurred < 24 months later. Immunocompromise and the presence of other underlying comorbidities were associated with increased severity, and women reported longer duration of illness.

**Disclosures:**

Moe H. Kyaw, PhD, Pfizer: Employee Catherine Martin, PhD, Pfizer: employee|Pfizer: Stocks/Bonds (Private Company) Maria Major, B.Sc., M.P.H., Pfizer: Employee Samira Mubareka, MD, Pfizer: Grant/Research Support Srinivas Valluri, PhD, Pfizer: Employee John M. McLaughlin, PhD, Pfizer: Employee|Pfizer: Stocks/Bonds (Public Company) Allison McGeer, MD, AstraZeneca: Honoraria|GSK: Honoraria|Merck: Honoraria|Moderna: Honoraria|Novavax: Honoraria|Pfizer: Grant/Research Support|Pfizer: Honoraria|Roche: Honoraria|Seqirus: Grant/Research Support|Seqirus: Honoraria

